# UPRIGHT, a resilience-based intervention to promote mental well-being in schools: study rationale and methodology for a European randomized controlled trial

**DOI:** 10.1186/s12889-019-7759-0

**Published:** 2019-10-29

**Authors:** Carlota Las Hayas, Irantzu Izco-Basurko, Ane Fullaondo, Silvia Gabrielli, Antoni Zwiefka, Odin Hjemdal, Dora G. Gudmundsdottir, Hans Henrik Knoop, Anna S. Olafsdottir, Valeria Donisi, Sara Carbone, Silvia Rizzi, Iwona Mazur, Anna Krolicka-Deregowska, Roxanna Morote, Frederick Anyan, Mette Marie Ledertoug, Nina Tange, Ingibjorg Kaldalons, Bryndis Jona Jonsdottir, Ana González-Pinto, Itziar Vergara, Nerea González, Javier Mar Medina, Esteban de Manuel Keenoy, on behalf of the UPRIGHT consortium, Maider Mateo, Igor Larrañaga, Iñaki Zorrilla, Patricia Pérez Martínez, Rosa Maimone, Solveig Karlsdottir, Sigrun Danielsdottir, Alda Ingibergsdottir, Hrefna Palsdottir, Unnur B. Arnfjord

**Affiliations:** 1 Kronikgune Institute for Health Services Research, Torre del BEC, Ronda de Azkue 1, 48902 Barakaldo, Bizkaia, Basque Country Spain; 2 Bruno Kessler Foundation, Trento, Italy; 3 Lower Silesia Voivodeship Marshal Office, Wrocław, Poland; 4 Norwegian University of Science and Technology, Trondheim, Norway; 5 Directorate of Health in Iceland, Reykjavík, Iceland; 6 Aarhus University, Aarhus, Denmark; 7 University of Iceland, School of Education, Reykjavík, Iceland; 8 Daily Centre for Psychiatry and Speech Disorders, Wrocław, Poland; 9 Wroclaw Medical University, Wrocław, Poland; 10 University Hospital Alava-Santiago, Spanish Society of Biological Psychiatry (CIBERSAM), Vitoria, Spain; 11 Research Unit. AP-OSIs Gipuzkoa, Mondragón, Spain; 12 Hospital Galdakao-Usansolo, Health Services Research on Chronic Patients Network- REDISSEC, Galdakao, Spain; 13 Clinical Management Unit, Hospital Alto Deba, Mondragón, Spain

**Keywords:** Adolescence, Resilience, Whole school approach, Health-promoting school, Mental health education, Mental disorders, Mental well-being

## Abstract

**Background:**

Adolescence is crucial period for laying the foundations for healthy development and mental well-being. The increasing prevalence of mental disorders amongst adolescents makes promotion of mental well-being and prevention interventions at schools important. UPRIGHT (Universal Preventive Resilience Intervention Globally implemented in schools to improve and promote mental Health for Teenagers) is designed as a whole school approach (school community, students and families) to promote a culture of mental well-being and prevent mental disorders by enhancing resilience capacities. The present article aims at describing the rationale, conceptual framework, as well as methodology of implementation and evaluation of the UPRIGHT intervention.

**Methods:**

UPRIGHT project is a research and innovation project funded by the European Union’s Horizon 2020 Research and Innovation programme under grant agreement No. 754919 (Duration: 48 months). The theoretical framework has been developed by an innovative and multidisciplinary approach using a co-creation process inside the UPRIGHT Consortium (involving seven institutions from Spain, Italy, Poland, Norway, Denmark, and Iceland). Resulted is the UPRIGHT programme with 18 skills related to 4 components: Mindfulness, Coping, Efficacy and Social and Emotional Learning.

Among the five Pan-European regions, 34 schools have been currently involved (17 control; 17 intervention) and around 6000 adolescents and their families are foreseen to participate along a 3-year period of evaluation. Effectiveness of the intervention will be evaluated as a randomized controlled trial including quantitative and qualitative analysis in the five Pan-European regions representative of the cultural and socioeconomic diversity. The cost-effectiveness assessment will be performed by simulation modelling methods.

**Discussion:**

We expect a short- to medium-term improvement of mental well-being in adolescents by enhancing resilience capacities. The study may provide robust evidence on intrapersonal, familiar and social environmental resilience factors promoting positive mental well-being.

**Trial registration:**

ClinicalTrials.gov Identifier: NCT03951376. Registered 15 May 2019.

## Background

Over the past decade, it has been an increasing incidence of mental health disorders in adolescents, which has grown into a global health burden [1]. The most prevalent forms of psychiatric disorders are anxiety, behavioural and mood disorders, and substance abuse [2]. Among psychiatric disorders in adults, those that have their onset in childhood tend to be more severe [3].

Thus, childhood and adolescence are sensitive periods in which mental health may be positively modifiable. Integrated school interventions focusing on adolescents offer the possibility of influence for a healthy development and decrease risk to develop a mental health disorder. Risk factors in adolescents associated with mental health problems mainly include social isolation, family conflict, stressful life events, emotional immaturity, academic failure, low self-esteem and poor body image [4], as well as health risk behaviours such as drug and alcohol use [5]. However, not all youth experiencing adversity or exposure to risk factors show negative mental health outcomes. The concept of resilience may be the key. Resilience is the ability of an individual or community to adapt to life challenges or adversities while maintaining mental health and well-being [6, 7]. Increasing resilience skills lead to lasting beneficial effects on a range of educational, social and economic outcomes, thus may prevent the development of mental health problems in adolescents [8, 9].

Interventions for mental health promotion adopting social and emotional learning (SEL) programmes have been conducted among others in the USA [10, 11], Australia [12, 13], Europe [14] and the UK [15], as a whole school approach collaboratively working with the school community, students and their families. Experience showed that these initiatives have supported positive mental health, reduced risky behaviour, at the time that raised academic attainment [16–19] or diminished suicide risk [14]. However, there is a clear need to increase evidence on effective interventions promoting mental well-being [20]; improving implementation strategies, or focus on identifying relevant training components and SEL curricula [13, 21, 22].

The UPRIGHT intervention, Universal Preventive Resilience Intervention Globally Implemented In Schools To Improve And Promote Mental Health For Teenagers, has been created to respond to the European Commission framework programme for research and innovation (Horizon 2020) related to the Work Programme 2016–2017 on “Health, demographic change and well-being”, and specifically promoting mental health and well-being in the young (SC1-PM-07–2017) [20]. Promoting resilience by training in the UPRIGHT programme requires an ecological view by tackling school environment, the school staff, family and adolescents themselves and by fostering a broad range of interactive protective factors in each focus participant group.

### Objectives of UPRIGHT

UPRIGHT general aim is to promote mental well-being and prevent mental disorders in youth by enhancing resilience capacities, through a whole school approach addressing early adolescents, their families and the school community, to create a real mental well-being culture at schools.

The operational objectives of UPRIGHT are: i) to co-create (involving adolescents, families, school staff, clinicians, policy makers) an innovative holistic resilience programme in schools for the promotion of mental health in youth between 12 and 14 years; ii) to deploy an intervention in five different pan-European regions; iii) to better understand the natural history of mental disorders according to the resilience level and provide evidence of specific resilience factors promoting positive mental well-being longitudinally; iv) to demonstrate the effectiveness and predict future impact of an intervention in terms of improvement of quality of life, mental well-being, and academic performance, and a reduction of absenteeism and bullying cases; and v) to transfer the programme to Europe and beyond by disseminating the results and enabling innovative action plans for mental well-being in the youth.

In the present article, we present the conceptual framework and co-creation process of the programme, as well as implementation resources in schools to reach a universal intervention adaptable to the particularities and mental health needs in the five participating pan-European regions. As a trialled intervention, UPRIGHT initiative will be under evaluation during a 3-year period.

## Methods

### Design and ethics approval

Following the Standard Protocol Items: Recommendations for Interventional Trials (SPIRIT 2013 Statement) we provide information on the clinical trial procedures and methods in the SPIRIT diagram (Additional file 1) and the SPIRIT checklist (Additional file 2). The UPRIGHT Consortium is comprised by multidisciplinary professionals from 7 partners in a pan-European setting (Additional file 3). The UPRIGHT research project is a cluster, randomized, controlled (two parallel groups) trial expecting to involve nearly 6000 adolescents and their families in five regions, including Spain, Italy, Poland, Denmark and Iceland. The project was approved by the institutional review boards of the pilot regions. UPRIGHT researchers in collaboration with schools obtained signed informed consent forms from all participants, including teachers, families (legal tutors also signed consent forms for adolescents participation) and adolescents (12–14 years of age signed assent forms) before any study data was collated.

Schools that committed to participate in the project were stratified according to the number of children they have, their location (rural or other) and the socio-economic status. Then, block randomization was performed by statistical free software R v3.4.0 and schools were distributed to intervention or control groups. Control schools implemented usual curricula and were not provided intervention resources or support, with the exception of the questionnaires to evaluate study outcomes.

### UPRIGHT intervention

#### A resilience-based programme

The UPRIGHT conceptual framework was initially based on an extensive literature search regarding the existing resilience-based, mental health promotion interventions in schools. The expert committee comprised by mental health professionals (psychologists, psychiatrists, psycho-pedagogists) from seven European organizations within the UPRIGHT consortium (Additional file 3) has extensive experience on resilience and school based programmes. The expert committee defined the theoretical framework of the programme ensuring that skills important during the adolescence period were included, redundancy was avoided and operationability of the model was feasible to become implemented in schools.

**Fig. 1:**
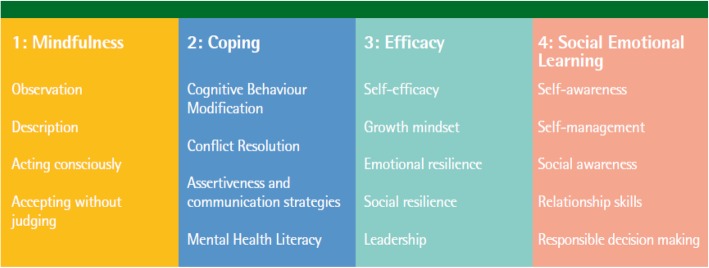
Theoretical framework with the main components and skills to increase resilience among adolescents

The final UPRIGHT programme comprised 4 components and 18 skills (Fig. 1):

**Coping**, as the ability of responding and managing challenges effectively [23]. The skills to train coping were cognitive behaviour modification, conflict resolution, assertiveness and communication strategies and mental health literacy.

**Efficacy**, as the confidence on having individual and social capabilities required to produce an outcome [24]. This component included materials on self-efficacy, growth mindset, emotional resilience, social resilience and leadership skills.

**SEL** that comprises the core competencies to handle interpersonal situations constructively (in short, they are lessons in emotional intelligence) [10]. The SEL construct was composed of the attributes of self-awareness, self-management, social awareness, relationship skills and responsible decision making.

**Mindfulness**, which was described to be a flexible state of mind [25], paying attention in a particular way: on purpose, in the present moment, and non-judgmentally [26]. Mindful practice on observation and description, acting consciously and accepting without judging was transversally presented throughout the three components of the programme.

#### Co-creation and regional adaptation of the UPRIGHT programme

The next step was to define co-creation innovative methods involving the young themselves in order to co-design, co-produce and co-customize the previously described UPRIGHT programme. According to their needs, resources and expectations, the intention was to ensure trustworthiness and relevance of the core intervention across European regions with a different cultural and socioeconomic backgrounds (Basque Country in Spain, Trento in Italy, Lower Silesia in Poland, Denmark and Reykjavik area in Iceland). The specific objectives for the co-creation and regional adaptation of the UPRIGHT programme are described in Table 1.

**Table 1:** Specific objectives for the co-creation and regional adaptation of the UPRIGHT programme

Objectives of the UPRIGHT co-creation and regional adaptation	
• To involve the young themselves and other relevant stakeholders gathering their inputs for the design of the intervention.	
• To confirm that the three groups of participants (adolescents, families, teachers/school staff) have a clear understanding of the four core components and 18 skills comprising the UPRIGHT theoretical model.	
• To prioritize the most relevant or meaningful resilience skills for everyday life according to the three groups of participants.	
• To identify and prioritize the most relevant areas of concern for adolescents’ development and mental health according to the three groups of participants.	
• To select and prioritize the most relevant and feasible methodologies to implement the UPRIGHT programme with youth, families, and school staff.	
• To identify collectively main challenges and needs (from the community, school, and families) for the successful implementation of UPRIGHT (and their possible solutions).	
• To identify collectively main resources and expectations in the community, school, and families for the successful implementation of UPRIGHT.	
• To explore the cultural context and antecedents for the implementation of UPRIGHT in the school: inclusion, active participation, positive relationships, belonging and mental health (school resilience).	
• To adapt and co-customize the UPRIGHT programme to regional needs and expectations in the five different European regions (pilot sites).	

The co-creation involved a mixed-methodology that combined participatory strategies, qualitative methods, and quantitative data collection. Participatory working group sessions and surveys were the two main research strategies that were independently planned with the three target groups (adolescents, families and teachers/school staff).

On the one side, participants meeting inclusion criteria and who signed informed consent form participated in the working group sessions. A specific protocol of activities and supplementary pedagogic materials was developed for each target group to perform the participatory activities. For instance, an adapted version of a participatory diagnostic tool ‘The problem tree analysis’ was directed to families and school staff, to evaluate the risks and possible solutions related to their participation in UPRIGHT and its implementation in the school setting.

On the other side, a survey was created ad-hoc to collect information on the co-creation objectives from a convenient sample of people from the three target groups. Also the surveys included items to evaluate the quality of the school climate. Items were responded using a Likert-type response scale as well as open-ended questions to collect suggestions and opinions from the participants.

The co-creation methods and outputs were subjected to an internal audit in order to provide an account of the validity and reliability of the procedures followed across sites. Appointed auditors from each of the five UPRIGHT pilot regions analysed methods and data gathered from the working groups and surveys. For the audit trail detailed qualitative protocols were used to analyse respondent and content validity, transparency, representability and plausibility. In addition, the programme content validity was explored asking one student, one teacher and one family from each country to review it. To do so, each validation-volunteer read some assigned chapters and marked how feasible (possible to do) and relevant (meaningful) the skill was for his/her daily life.

### UPRIGHT programme implementation in five pan-European regions

#### Conception of an adaptable but structured intervention. Implementation strategies

Engaged schools in UPRIGHT analysed the conceptual framework ensuring that it was aligned with their strategic priorities and that the methodology of implementation was feasible. At the time of this publication, the UPRIGHT resilience-based intervention is undergoing with a total of 34 committed schools participating in a pan-European setting, representing economic, sociodemographical and cultural diversity.

**Fig. 2:**
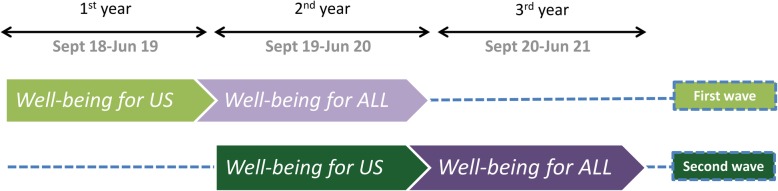
Scheme of implementation per European region participating in the UPRIGHT project

The intervention design consists of two different phases consecutively implemented: Well-being for US and Well-being for ALL (Fig. 2). During the first phase, Well-being for US, all stakeholders are trained in the UPRIGHT programme. The second phase, Well-being for ALL intends not only to maintain the effect of the intensive training in youths, but also to boost the positive mental health atmosphere created in the whole school. To do so, different collective activities will be organized at school level such as celebration of thematic days, activities with the community, and outdoor/indoor activities.

The UPRIGHT intervention (Well-being for US and Well-being for ALL) will be implemented twice (two waves) within the duration of the research project. Analysis of the UPRIGHT intervention in the first wave will serve to identify areas of improvement for the second wave. The two waves will last three school years (Fig. 2), meaning that the Well-being for ALL programme of the first wave and the Well-being for US of the second wave will be deployed at the same time (second school year).

**Fig. 3:**
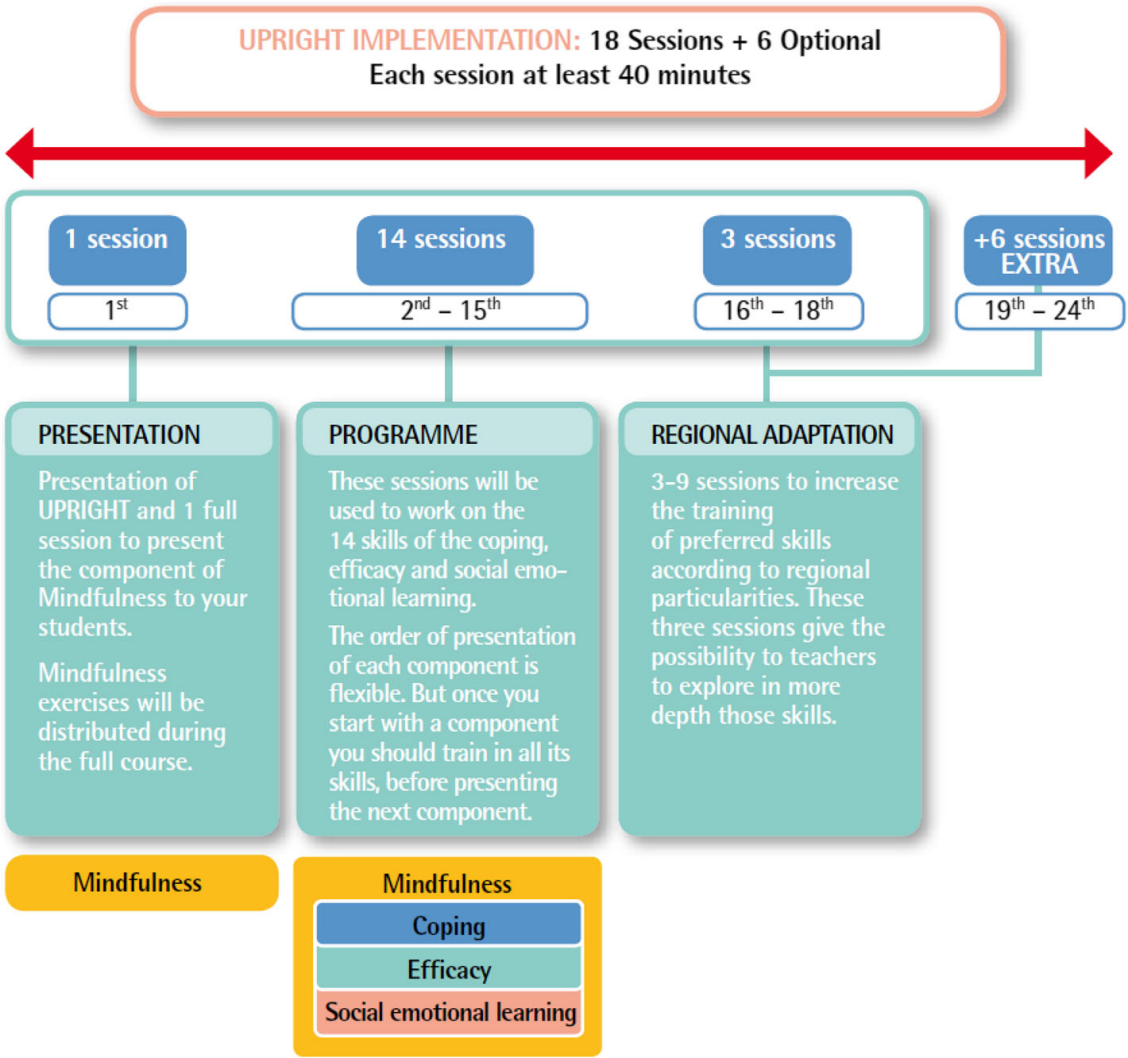
The prescribed implementation framework of the UPRIGHT programme across schools in 5 Pan-European regions

The UPRIGHT implementation was designed to integrate particularities of schools, including educational needs and local context. Nonetheless, a prescribed common framework (Fig. 3) will be warranted by monitoring visits to schools during the 3-year evaluation time of the intervention. UPRIGHT defined a minimum and maximum number of sessions that must be carried out with the students in order to ensure effectiveness (18 and 24 sessions, respectively; Fig. 3). The premise was that once the minimum sessions were covered, the extension of the training on UPRIGHT programme could be focused to reinforce those skills that better resembled particular school needs and regional preferences.

#### Training of the target groups

UPRIGHT intervention is primarily psychoeducational based on providing education by theory and dynamic exercises to develop resilience capacities. Teachers and school staff received the training via face-to-face group sessions organised by experienced UPRIGHT local trainers. Then teachers themselves trained adolescents on UPRIGHT skills in 18 to 24 group-sessions of at least 40 min each to be integrated as part of the daily life of the school. The families trained on the programme via the web platform and face-to-face training sessions (intended to give families an overview of the programme and promote their participation).

The relevant training materials and contents of the programme included the paper-based manual for teachers, short videos that illustrate skills and components and seven mindfulness audios, all created ad hoc for the intervention. All these materials are also displayed in intuitive formats in the web platform (www.uprightprogram.eu), which currently serves for families and teachers (password protected), and have a public space focused on the community to present social events related to UPRIGHT.

### A 3-year follow-up evaluation

The evaluation of the programme will be performed in two consecutive school years starting school year 2018–19 and 2019–20 (Fig. 2) with three repeated measures: baseline evaluation, starting at the beginning of the Well-being for US programme at each wave; midterm assessment, at the end of the Well-being for US phase; and final assessment carried out at the end of the Well-being for ALL phase at each wave.

#### Study outcomes assessments

Impact and effectiveness of the intervention on target groups (adolescents, families, and teachers/school staff) will be assessed using quantitative and qualitative methods. For the quantitative approach, self-reported questionnaires will be used for each target group. In adolescents, the quantitative evaluation will measure mental well-being, resilience factors, quality of life, stress perception, behaviour disorders (violence and bullying), mental disorders (depression, anxiety, and addictive disorders), and school resilience (Table 2). School records will serve to measure absenteeism of teachers and students, cases of conflicts/fights where a student was involved, academic achievements, school dropouts, and bullying cases. The effect of the UPRIGHT intervention over the course of the follow-up will be assessed with generalized mixed models for longitudinal data. These models will take into account the repeated measurements for each participant and also the clustered structure of the data.

**Table 2:** List of validated scales included in the self-reported questionnaires according to the UPRIGHT target groups

Outcome	Scale	Adolescents	Families	Teachers
Sociodemographic	Sociodemographic	√	√	√
Mental well-being	WEMWBS-14 [27]	√	√	√
Perceived stress	PSS-4 [28]	√	√	√
School resilience	NTNU *ad hoc scale*	√	√	√
Resilience for adolescents	READ-28 [29]	√		
Quality of life	Kidscreen-10 [30]	√		
Bullying, substance use, violence and injuries	HBSC sub-scales [31]	√		
Mental disorder - depression	PHQ-9 [32]	√		
Mental disorder - anxiety	GAD-7 [33]	√		
Resilience for adults	RSA-33 [34]		√	√
Cohesion and flexibility	FACES IV [35] sub-scales		√	

The use of qualitative methodology in UPRIGHT will aim to getting schools, youth and families explore the impact and effectiveness of the intervention and detail their experience with the whole process of implementation. The focus will be understanding their level of satisfaction and acceptability, as well as the self-perceived improvement of mental well-being. The qualitative analysis will cover two time points (follow-up and final) and will include semi-structured interviews with schools and families, and focus groups with youths.

#### Simulation modelling

As part of the health economic analysis, a Discrete Event Simulation model will be built to estimate the economic impact of the UPRIGHT intervention at the population level beyond the duration of the study. First, definition of the natural history of mental disorders among youths will be necessary. Second, the simulation model will be developed and fed using various information sources to define the current epidemiological scenario. Construction of a risk score will be based on the battery of questionnaires selected (Table 2). Then, the information from the questionnaires will be integrated in a unique score measuring the probability of developing mental disorders. Construction and validation of the simulation model that represents the current epidemiological scenario will lead to the estimation of the impact of the intervention at the population level by incorporating in the simulation model the results from the pilot site and the calculated risk score.

#### Sample size calculation

To obtain an effect size of 0.34 in the primary endpoint, improvement on well-being, sample size was calculated. A total of 5992 adolescents (2996 students per group) will be required in the five pilot regions to detect an improvement on mental health with a 80% of statistical power and significance level of 0.05.

## Expected impact

Once the UPRIGHT programme content was co-created and validated, the intervention was implemented in the schools from the five pan-European pilot regions starting on the school year 2018–19. At the time of this publication, baseline evaluation of the first wave analysis is ongoing. The assessment of the areas of improvement identified in the first wave is also running to introduce changes in the procedures for the second wave.

Overall, the UPRIGHT project expects a direct positive impact in the short-term mental well-being of 3000 youngs attending intervention schools. In addition, all students and education professionals in the UPRIGHT intervention schools (around 24,000 children from 6 to 16 years old) will benefit from the change of culture in the schools. Co-design, co-production and co-customisation of the UPRIGHT intervention will guarantee that diversity is preserved, avoiding any potential misunderstanding or rejection of the intervention by teachers/school staff, families and youths from different sites.

UPRIGHT aims to raise awareness of the consequences of bullying, discrimination and violence and expects to contribute in reducing its burden at schools, also including short-term dropouts. UPRIGHT preventive strategy will have an effect of reducing the occurrence of mental disorders as well as co-morbidities associated later in life. The programme will act and change factors that have a direct impact in mental health, such as unhealthy behaviour patterns, stress, anxiety and drug abuse.

## Discussion

Universal school-based interventions have great potential to target large populations of young people and promote cultures of positive mental health by involving school staff and families at a general level. UPRIGHT project ambition is to be the first school-based primary intervention programme that will promote mental health and well-being, targeting adolescents of 12–14 years of age.

Previous programmes have been developed, but measures of mental health determinants and outcomes were heterogeneous. Despite the benefits found in many areas involving mental well-being, their experience showed lack of effectiveness in some interventions due to barriers in the implementation and lack of engagement of school teachers [13, 19, 21, 22, 36, 37]. Schools appeared to welcome flexibility and autonomy [38], but unspecific guidelines and unclear instructions resulted in leaving schools confused and insufficient progress [39]. The UPRIGHT project has developed a flexible, but structured intervention of 18–24 sessions that will be inserted as part of the school curricula for adolescents. A manual for the teachers and an online platform with useful materials have been created ad hoc. The best components of these interventions are also to be determined [21]. All ingredients in the conceptual framework of UPRIGHT programme were defined based on the previous experience and thereafter rated as feasible and relevant among the involved countries and groups of participants (co-creation methodology).

The development of a two-wave intervention will permit the UPRIGHT researchers to extend the period of implementation. On the one hand, essential to learn on the experience from the first wave an adapt/improve, if necessary, the content of the programme and procedures during the second wave implementation at schools. On the other hand, to prolong the evaluation, which will lead on providing enough evidence on effectiveness and economic impact on the long run of the intervention. Other programmes tended to have limited intervention periods, weak and short term evaluation methodologies [36]; usually they ran for no more than 6 months. As a result, some implementation procedures have been described in the literature to be insufficient to ensure significant improvement in the mental health outcomes [36].

Other strength of having a co-created and validated programme is that the UPRIGHT intervention maybe extensible to other European countries and beyond, which is one of the common limitations of other programmes that lack of generalizability to other contexts and cultures. The economic impact of the UPRIGHT intervention will be aimed beyond the duration of the study as part of the health economic analysis by the use of modelling. It is critical to highlight whether investment in school-based mental health prevention and promotion interventions might represent good value to avoid future costs of poor mental health in healthcare and other public sector budgets [40]. This way we would be in conditions to reverse the current fact that mental health promotion may not be seen as a high priority for policy makers.

In conclusion, UPRIGHT ambition is to go beyond the limitations described by previous researches. It will build on the knowledge, experience and results of these programmes, with special emphasis to address the main limitations that have been described.

## Supplementary information

**Additional file 1.** SPIRIT diagram. The list of procedures for clinical trials.

**Additional file 2.** SPIRIT checklist. According to the recommendations for clinical trials, the SPIRIT checklist includes information on objectives, methodology etc. of the research Project.

**Additional file 3.** The UPRIGHT Consortium. The map of the Consortium and the list of institutions participating in the UPRIGHT project is provided in this file. List of the researchers included in the “on behalf of the UPRIGHT Consortium” term is also provided.

## Data Availability

Not applicable. The UPRIGHT consortium has the commitment with the European Commission to share study datasets (except that identifying/confidential patient data) in publicly available repositories. We are still working in the way we are going to make these data available (type of data and platform). The project is ongoing and datasets have not yet been completed, which is expected for the end of year 2021.

## Abbreviations

FACES: Family Adaptability and Cohesion Evaluation Scales
GAD: General Anxiety Disorder
HBSC: Health Behaviour in School-Aged Children
NTNU: Norwegian University of Science and Technology
PHQ: Patient Health Questionnaire
PSS: Perceived Stress Scale
READ: Resilience Scale for Adolescents
RSA: Resilience Scale for Adults
SEL: Social and emotional learning
UPRIGHT: Universal Preventive Resilience Intervention Globally implemented in schools to improve and promote mental Health for Teenagers
