# Supplementary material for: UPRIGHT, a resilience-based intervention to promote mental well-being in schools: study rationale and methodology for a European randomized controlled trial
Source: BMC Public Health. 2019 Oct 29;19:1413. doi: 10.1186/s12889-019-7759-0 (PMC6820972; doi:10.1186/s12889-019-7759-0)
Supplement: Supplementary file 1 — Additional file 1. SPIRIT diagram. The list of procedures for clinical trials. [file 12889_2019_7759_MOESM1_ESM.doc]

Figure. Example template of recommended content for the schedule of enrolment, interventions, and assessments.*

|  | **STUDY PERIOD** | | | | | | | |
| --- | --- | --- | --- | --- | --- | --- | --- | --- |
|  | **Enrolment** | **Allocation** | **Post-allocation** | | | | | **Close-out** |
| **TIMEPOINT**** | ***-t_1_*** | **0** | ***t_1_*** | ***t_2_*** | ***t_3_*** | ***t_4_*** | ***etc.*** | ***t_x_*** |
| **ENROLMENT:** |  |  |  |  |  |  |  |  |
| **Eligibility screen** | X |  |  |  |  |  |  |  |
| **Informed consent** | X |  |  |  |  |  |  |  |
| ***[List other procedures]*** | X |  |  |  |  |  |  |  |
| **Allocation** |  | X |  |  |  |  |  |  |
| **INTERVENTIONS:** |  |  |  |  |  |  |  |  |
| ***[Intervention A]*** |  |  |  |  |  |  |  |  |
| ***[Intervention B]*** |  |  | X |  | X |  |  |  |
| ***[List other study groups]*** |  |  |  |  |  |  |  |  |
| **ASSESSMENTS:** |  |  |  |  |  |  |  |  |
| ***[List baseline variables]*** | X | X |  |  |  |  |  |  |
| ***[List outcome variables]*** |  |  |  | X |  | X | etc. | X |
| ***[List other data variables]*** |  |  | X | X | X | X | etc. | X |

*Recommended content can be displayed using various schematic formats. See SPIRIT 2013 Explanation and Elaboration for examples from protocols.

**List specific timepoints in this row.
