# Supplementary material for: UPRIGHT, a resilience-based intervention to promote mental well-being in schools: study rationale and methodology for a European randomized controlled trial
Source: BMC Public Health. 2019 Oct 29;19:1413. doi: 10.1186/s12889-019-7759-0 (PMC6820972; doi:10.1186/s12889-019-7759-0)
Supplement: Supplementary file 2 — Additional file 2. SPIRIT checklist. According to the recommendations for clinical trials, the SPIRIT checklist includes information on objectives, methodology etc. of the research Project. [file 12889_2019_7759_MOESM2_ESM.pdf]

SPIRIT 2013 Checklist: Recommended items to address in a clinical trial protocol and related documents*

Section/item	Item No	Description
**Administrative information**		
Title	1	"Title, page 1, paragraph 1"<br>UPRIGHT, A RESILIENCE-BASED INTERVENTION TO PROMOTE MENTAL WELL-BEING IN SCHOOLS: STUDY RATIONALE AND METHODOLOGY FOR A EUROPEAN RANDOMIZED CONTROLLED TRIAL
Trial registration	2a	"Abstract; page 3, paragraph 2"<br>ClinicalTrials.gov Identifier: NCT03951376.
Protocol version	3	Not applicable
Funding	4	"Declarations, page 18; under subheading *Funding*"<br>UPRIGHT is a research and innovation project funded by the European Union´s Horizon 2020 Research and Innovation programme under grant agreement No. 754919. This paper reflects only the authors' views and the European Union is not liable for any use that may be made of the information contained therein. The funding body has had no role in the study design, in the writing of the protocol or in the decision to submit the paper for publication.
Roles and responsibilities	5a	List of authors and affiliations: Title page; page 1; paragraph 2"<br>Contributions: "Declarations, page 18; under subheading *Authors' contributions*"
	5b	"Declarations, page 18; under subheading *Authors' contributions*"<br>Esteban de Manuel Keenoy (EMK). Kronikgune Institute for Health Services Research, Basque Country (Spain).
	5c	"Declarations, page 18; under subheading *Authors' contributions*"<br>EMK participated in the design and coordination of the study at the European level and in the critical review of the manuscript.<br><br>"Declarations, page 18; under subheading *Funding*"

	5d	**Coordinators**:<br>Esteban de Manuel Keenoy. Coordinator of the project.<br>Ane Fullaondo. Deputy of the coordinator.<br>Carlota Las Hayas. Principal investigator.<br>Irantzu Izco-Basurko. Project manager.

Steering committee:

Esteban	De Manuel Keenoy	Kronikgune Institute for Health Services Research, Basque Country (Spain).
Ane	Fullaondo	Kronikgune Institute for Health Services Research, Basque Country (Spain).
Irantzu	Izco-Basurko	Kronikgune Institute for Health Services Research, Basque Country (Spain).
Hans Henrik	Knoop	Aarhus University, Aarhus (Denmark).
Odin	Hjemdal	Norwegian University of Science and Technology, Trondheim (Norway).
Silvia	Gabrielli	Bruno Kessler Foundation, Trento (Italy).
Antoni	Zwiefka	Lower Silesia Voivodeship Marshal Office (Poland).
Anna Sigríður	Ólafsdóttir	University of Iceland, School of Education, Reykjavík (Iceland).
Dóra Guðrún	Guðmundsdóttir	Directorate of Health in Iceland, Reykjavík (Iceland).

Introduction

Background and rationale	6a	“Background, pages 4 and 5”.
	6b	Not applicable as described in “Methods; page 6; under subheading *Design and ethics approval*; paragraph 2”<br>The intervention is compared with the common practice at schools.
Objectives	7	“Background; under subheading *Objectives of UPRIGHT*; pages 5 and 6”
Trial design	8	“Methods; page 6; under subheading *Design and ethics approval*; paragraph 1”.

Methods: Participants, interventions, and outcomes

Study setting	9	“Methods; page 6; under subheading *Design and ethics approval*; paragraph 1” and additional file 1.
Eligibility criteria	10	“Methods; page 6; under subheading *Design and ethics approval*; paragraph 2”.

Interventions	11a	**Intervention group**: "Methods; under subheading *2. UPRIGHT intervention*; pages 6-9.<br>**Control group**: "Methods; page 6; under subheading *Design and ethics approval*; paragraph 2".
	11b	Not applicable, this is a behaviour intervention and it is not expected to arm or disturb the daily life of participants. In case any problem is detected in adolescents during the intervention, teachers or families will follow the procedures as per school protocols and regulation applicable at each region.
	11c	Not applicable (no drug involving intervention).
	11d	The trial is implemented in a real-life setting thus allowing other psycho-educative programmes and behaviour interventions can be in place.
Outcomes	12	Primary: Change in mental well-being and the change in resilience capacities for adolescents.<br>Secondary: Change in resilience capacities for adults and school resilience capacities; change in perceived stress (PSS); Change in the quality of life of adolescents (Kidscreen-10); change in the cases of bullying, substance use, violence and injuries (HBSC); change in the incidence of anxiety (GAD-7); and change in the incidence of depression (PHQ9).
Participant timeline	13	"Methods; under subheading *2. UPRIGHT programme implementation in five pan-European regions*; page 10; paragraphs 2 and 3.
Sample size	14	"Methods; under subheading *Sample size calculation*; page 13; paragraph 1".
Recruitment	15	The commitment of the school managers is essential to reach families. The teachers and adolescents will be recruited from the schools enrolled.

Methods: Assignment of interventions (for controlled trials)

Allocation:		
Sequence generation	16a	"Methods; page 6; under subheading *Design and ethics approval*; paragraph 2".
Allocation concealment mechanism	16b	"Methods; page 6; under subheading *Design and ethics approval*; paragraph 2".
Implementation	16c	Not applicable. The schools are randomized, not participants.
Blinding (masking)	17a	Not applicable.
	17b	Not applicable.

Methods: Data collection, management, and analysis

Data collection methods	18a	"Methods; under subheading *3. A 3-year follow-up evaluation*; page 11"<br>List of validated questionnaires: "Methods; under subheading *Study outcomes assessments*; table 2"
	18b	All participants (adolescents, teachers and families) will be invited to complete the questionnaires in the tree time points. Reminders will be sent by email (families) and phone call (school) in order to include the maximum number of participants. Despite that the programme is online for families, several face-to-face meeting will be conducted to engage their participation.
Data management	19	A codebook was developed in Excel, describing all the variables, type of data, as well as validation rules, to facilitate data collection. According to this, then, pseudonymized data from each pilot site was encoded into csv templates by a simple excel database.<br>A quality control of the data was performed by the evaluation team (high frequency of missing values, missing data for mandatory or important outcomes, values considered being unrealistic outliers, quantitative variables falling outside the min-max range, categorical variables not related to the predefined categories, impossible or not adequate dates).
Statistical methods	20a	Baseline evaluation will include a descriptive analysis for all variables included in the study as well as the differences observed between arms. The effect of the intervention over the course of the follow-up will be assessed with generalized mixed regression models for longitudinal data. These models will take into account the repeated measurements for each participant and also the clustered structure of the data, these will be included as random effect. Study arm, time of measurement and the interaction between them will be included as fixed effects in the models. Participants and schools will be included as random effects. Models will be adjusted for potentially influential factors. Statistical analyses will be carried out using the free software R v.3.4.0 or higher.
	20b	"Methods; under subheading *Study outcomes assessments*; page 13; paragraph 1" (Qualitative methodologies) and "Methods; under subheading *Predictive modelling*; page 13".
	20c	Analyses will be carried out on an intention-to-treat basis. The available data of all patients initiating the study will be assessed as part of their allocation group, even for the lost to follow up cases.
**Methods: Monitoring**		
Data monitoring	21a	A data manager from each pilot regions is nominated, who will be responsible of the data management and processing. The data and safety management team will be formed by members of the UPRIGHT consortium. A Data Protection Officer (DPO) has been also nominated and he is independent from the coordinator of UPRIGHT. This DPO will work closely with Data and Safety Management Group.

	21b	An interim analysis will be done after the baseline data collection and then after the midterm evaluation. Analysis of data from the first wave will allow the UPRIGHT consortium to decide whether the project needs any amendment or not.
Harms	22	This is a behavioural intervention and thus no damage is expected in participants. However, in case adolescents feel bad or families identify any other discomfort, the communication of these events will be by following schools' procedures and according to regulations applicable at each region.
Auditing	23	The training of the participants in the UPRIGHT programme will be monitored by making visits to the schools (at least two per school year). Registration of the information is collated in the templates developed for monitoring the intervention.
**Ethics and dissemination**		
Research ethics approval	24	"Declarations; under subheading *Ethics approval and consent to participate*; page 17"
Protocol amendments	25	In case a relevant change is needed, first agreement by the entire UPRIGHT consortium is needed and a signature of a new version of the grant agreement. Then, the new grant agreement will be submitted to the European Commission via the project officer and if accepted, to the ethical committees at each pilot site. The changes will be finally transmitted to the schools' managers in order to ensure the optimal implementation.
Consent or assent	26a	After approval of the informed consent form by the ethical committees, explanation of the information and signature of the participants (families and teachers) will be asked by the UPRIGHT data managers at each pilot region. In the case of adolescents, their families or legal guardians will sign the consent and the adolescent him/her-self will also be asked to confirm their participation (they are 12 years old or over) by signing an assent form via the school managers.
	26b	Not applicable.
Confidentiality	27	The confidential information will be stored in safe in the UPRIGHT files and will not be shared with any other third institution or entity, except in response to a legal requirement. The data will be pseudonomized and submitted to the evaluation team for analysis. Only the data manager will have access to all personal data at each pilot region. A risk evaluation has been done in some of the regions, according to the applicable local regulations.
Declaration of interests	28	The investigators declare no financial or other competing interests.
Access to data	29	"Declarations; under subheading *Availability of data and materials*; pages 17 and 18".

Ancillary and post-trial care	30	No harm or damage in expected from the behaviour intervention under study. This section does not apply to the project.
Dissemination policy	31a	The dissemination plan includes several open access scientific articles, conferences in congresses and events, but also short articles and news in the website of the project; posts in social media and meetings with schools managers to present the project status.
	31b	The UPRIGHT consortium has agreed and created a Publication Board and a Publication Policy. The last is a document including description of the procedures in relation to any dissemination activity under UPRIGHT project (articles, conferences, social media and mass media (newspapers, TV news, radio interviews, etc.).
	31c	The horizon2020 research and innovation projects are mostly open via the Funding & Tender portal (https://ec.europa.eu/info/funding-tenders/opportunities/portal/screen/home). Procedures described in the deliverables and other documents will be mostly public after revision of the project officer.
**Appendices**		
Informed consent materials	32	The informed consent forms has been created in the local languages (Spanish, Basque, Italian, Polish, Danish and Icelandic) to be approved by the local ethical committees, thus different versions according to local regulation are currently in place.
Biological specimens	33	No biological specimens for genetic or molecular analysis are currently collected in the current trial. Not applicable.

*It is strongly recommended that this checklist be read in conjunction with the SPIRIT 2013 Explanation & Elaboration for important clarification on the items. Amendments to the protocol should be tracked and dated. The SPIRIT checklist is copyrighted by the SPIRIT Group under the Creative Commons "Attribution-NonCommercial-NoDerivs 3.0 Unported" license.
