# Supplementary material for: UPRIGHT, a resilience-based intervention to promote mental well-being in schools: study rationale and methodology for a European randomized controlled trial
Source: BMC Public Health. 2019 Oct 29;19:1413. doi: 10.1186/s12889-019-7759-0 (PMC6820972; doi:10.1186/s12889-019-7759-0)
Supplement: Supplementary file 3 — Additional file 3. The UPRIGHT Consortium. The map of the Consortium and the list of institutions participating in the UPRIGHT project is provided in this file. List of the researchers included in the “on behalf of the UPRIGHT Consortium” term is also provided. [file 12889_2019_7759_MOESM3_ESM.docx]

**The UPRIGHT Consortium**

^©^The UPRIGHT project. All rights reserved.

Map image sourced from [Venngage](https://venngage.com/) Inc. (<https://venngage.com/>)

**The UPRIGHT coordinator, partners and third parties.**

| **Project Coordinator** | | |
| --- | --- | --- |
| Spain | Kronikgune - Institute for health services research | **KRONIKGUNE** |

| **UPRIGHT Consortium Partners** | | |
| --- | --- | --- |
| Italy | Fondazione Bruno Kessler | **FBK** |
| Poland | Lower Silesia Voivodeship Marshal Office | **UMWD** |
| Norway | Norges Teknisk-Naturvitenskapelige Universitet | **NTNU** |
| Iceland | Directorate of Health in Iceland | **DOHI** |
|  | University of Iceland | **UoI** |
| Denmark | Aarhus University | **AU** |

| **Third Parties** | | |
| --- | --- | --- |
| Spain | Servicio Vasco de Salud - Osakidetza | **Osakidetza** |
| Poland | Daily Centre for Psychiatry and Speech Disorders | **Interia** |
| Poland | A. Falkiewicz Specialist Hospital | **Falkiew** |

**“on behalf of the UPRIGHT Consortium”** includes the following co-authors**:**

| **Beneficiary** | **Country** | **Author** | **Email** |
| --- | --- | --- | --- |
| **Kronikgune** | **Spain** | Maider Mateo | MAIDER.MATEOABAD@osakidetza.eus |
|  |  | Igor Larranga | Igor.LarranagaUribetxebarria@osakidetza.eus |
| **Osakidetza** | **Spain** | Inaki Zorilla | inaki.zorrillamartinez@osakidetza.eus |
|  |  | Patricia Pérez Martínez | patricia.perezmartinezdearrieta@osakidetza.eus |
| **FBK** | **Italy** | Rosa Maimone | rmaimone@fbk.eu |
| **DOHI** | **Iceland** | Solveig Karlsdottir | solveig@landlaeknir.is |
|  |  | Sigrun Danielsdottir | sigrun@landlaeknir.is |
| **UoI** | **Iceland** | Alda Ingibergsdottir | aldai@hi.is |
|  |  | Hrefna Palsdottir | hrefnapals@hi.is |
|  |  | Unnur B. Arnfjord | ubj@hi.is |
